# Foregone Health Care During the COVID-19 Pandemic: Early Survey Estimates from 39 Low- and Middle-Income Countries

**DOI:** 10.1093/heapol/czac024

**Published:** 2022-03-11

**Authors:** Jakub Jan Kakietek, Julia Dayton Eberwein, Nicholas Stacey, David Newhouse, Nobuo Yoshida

**Affiliations:** The World Bank; The World Bank; Department of Health Policy, London School of Economics and Political Science; The World Bank; The World Bank

**Keywords:** Foregone healthcare, COVID-19, LMICs

## Abstract

In addition to the direct health effects of the COVID-19 pandemic, the pandemic has increased the risks of foregone non-COVID-19 health care. Likely, these risks are greatest in low- and middle-income countries (LMICs), where health systems are less resilient and economies more fragile. However, there are no published studies on the prevalence of foregone health care during the pandemic in LMICs. We used pooled data from phone surveys conducted between April and August 2020, covering 73,638 households in 39 LMICs. We estimated the prevalence of foregone care and the relative importance of various reported reasons for foregoing care, disaggregated by country-income group and region. In the sample, 18·8% (95% CI 17·8%–19·8%) of households reported not being able to access health care when needed. Financial barriers were the most-commonly self-reported reason for foregoing care, cited by 31·4% (28·6%–34·3%) of households. More households in wealthier countries reported foregoing care for reasons related to COVID-19 ((27·2% [22·5%–31·8%] in upper middle-income countries compared to 8·0% [4·7%–11·3%] in low-income countries); more households in poorer countries reported foregoing care due to financial reasons (65·6% [59·9%–71·2%]) compared to 17·4% [13·1%–21·6%] in upper middle-income countries. A substantial proportion of households in LMICs had to forgo health care in the early months of the pandemic. While in richer countries this was largely due to fear of contracting COVID-19 or lockdowns, in poorer countries foregone care was due to financial constraints.

## Introduction

As of May 2021, there have been over 2 million COVID-19 deaths and 93 million confirmed COVID-19 cases in low and middle-income countries (LMICs) – about 56% of the total global burden of mortality and 54% of morbidity from the disease.(1) In addition to its direct impact, the pandemic has increased the risk of foregone non-COVID-19 care.(2) There are concerns that many individuals may choose not use health services because of fear of being exposed to COVID-19 at health care facilities, because of movement and other limitations imposed by national lockdowns, and due to financial constraints resulting from the economic downturn. The pandemic may have also negatively affected the supply of health services by overwhelming health facilities with large numbers of COVID-19 patients, diverting the use of resource from essential health services to COVID-19 management and care, or disrupting supply chains for essential commodities and equipment. Those demand and supply risks are particularly elevated in LMICs, where health systems are less resilient and economies more fragile. During the 2014-2015 Ebola outbreak average health care utilization declined by 18%, with even larger declines for maternal and child health services.(3-5) Modeling studies have shown that foregone utilization of essential health services may lead to substantial increases in mortality and morbidity.(6)

Emerging evidence from developed countries suggest that the utilization of some essential services declined during the COVID-19 pandemic, and recent studies have shown increased incidence of foregone care in the US and the European Union.(7-9) However, despite greater vulnerability of households and fragility of the health systems, there are no published studies on the prevalence of foregone health care during the pandemic in LMICs. WHOs pulse surveys of key respondents carried out in August 2020 and April 2021 presented data on perceptions of health sector government officials of the disruption in the provision of health services, but provided no quantitative data on the prevalence of foregone care or the types of barriers to utilization service users face.(10) A systematic review of literature found that maternal and fetal outcomes have worsened during the pandemic, but it included data from only two LMICs (Turkey and Nepal).(11) In contrast, a study of women's need for and use of contraceptives in Burkina Faso, Democratic Republic of Congo, Kenya, and Nigeria during the COVID-19 pandemic relative to the pre-COVID-19 period found mixed results: the need for contraception increased in Nigeria, and contraceptive use increased in Kenya and rural Burkina Faso.(12) None of the studies directly assessed whether individuals or households forewent the care they needed because of reasons directly related to COVID-19 or other reasons.

This study fills a critical evidence gap by providing estimates of foregone care during the early months of the pandemic in 39 LMICs, which account for 24.6% of the total population of LMICs, and 43.1% excluding India and China. Based on phone survey data from over 73,000 households and collected using a largely harmonized questionnaire and methods to assess foregone care, the data allows to produce estimates of the prevalence of foregone care and the prevalence of reasons for foregoing care across country income groups and regions. While aimed at improving understanding of foregone care during the pandemic, the analyses also constitute an important contribution to the general literature on foregone care in LMICs.

## Methods

*Data*

This study uses pooled household survey data from 39 high-frequency phone-based surveys conducted between April and August 2020 as part of the World Bank's initiative to monitor the socio-economic impact of COVID-19.(13) Annex Table 1 presents a list of countries and the month of survey data collection. The samples were drawn in three main ways. In 16 countries, the sample was drawn from a recent large in-person population survey, such as the Living Standard Measurement Survey. In another 16 cases, the samples were selected through random digit dialing (RDD). In another 7 cases, the samples were drawn from a pre-existing list of phone numbers. The surveys were designed to be representative of the target group at the national level. To correct for the bias resulting from non-random ownership of phones, in each survey, sampling weights were developed to adjust for the likelihood of the respondent household owning a phone and other household characteristics. Information was collected from one respondent per household, usually the household head, who respondent on behalf of the entire household.

*Outcome measures*

A generic questionnaire served as the basis for each country's survey, but in most cases some modifications were made to fit the local contexts. All surveys asked three questions related to the utilization of health services: 1.) whether the respondent or any member of their household needed medical care during the recall period (usually, 30 days); 2.) whether the respondent/household member was able to access the services they needed; 3.) if not, what was the main reason the respondent/household member could not access the services they needed. In most surveys, this last question was posed as an open-ended question, with the survey enumerator categorizing the answer using pre-identified categories. The categories included in most surveys were: financial barriers - (i) lack of money; (ii) lack of transportation; reasons directly related to COVID-19 - (iii) fear of contracting COVID-19; (iv) movement restrictions/lockdown/stay-at-home orders; reasons related to the supply of health services - (v) no medical personnel available at health facility; (vi) turned away because facility was full; and (vi) other [specify]. Some surveys included additional categories (e.g. visiting a traditional healer). Those were combined with the "other" category. For simplicity, in the analyses presented below, the reasons for forgoing care were grouped into four categories: 1.) financial barriers (lack of money; lack of transportation); 2.) lockdowns and fear of COVID; 3.) reasons related to the supply of health services (no medical personnel available; facility was full); and 4.) other reasons. Results disaggregated by each of the seven categories listed above are available in the Annex Table 2.Consistent with the literature, household were considered to forego care when they reported that they needed care and that they could not access the care they needed. Prevalence of foregone care was calculated as the households who could not access care as the percentage of the households reporting needing care.

*Statistical analysis*

The statistical analysis is descriptive in nature and focuses on documenting the extent of reported foregone care, reasons for households foregoing care, and differences in those indicators among countries from different regions and income groups. Data from the 39 surveys were pooled into a single data set. Point estimates and 95% confidence intervals are reported for the pooled sample and by country income group and global region. In addition, large sample two-sample tests of proportion equivalence were carried out to compare low-income countries with (i) lower-middle income countries and (ii) upper-middle income countries, and Sub-Saharan African countries with (i) East-Asia and Pacific countries, (ii) Latin American and Caribbean countries, and (iii) Middle East and North African countries. For pooled sample analysis, household sampling weights were adjusted by the country's population as a proportion of the population of all countries included in the study. For income group and region estimates, sampling weights for observations of each country were adjusted by their country's population as a proportion of the total population of the countries in the grouping, so that each country's contribution to the average was proportional to its population (see Annex for further detail).

*Contextual Measures*

To provide further context to the estimates of foregone care prevalence, we construct measures of COVID burden, lockdown stringency at the country income group level. These are the average of the number of total cases per million in the countries included in the income group (sourced from Dong et al. (1)), weighted by country population. Similarly, we construct a population-weighted average of the lockdown stringency index of Hale et al. (14) for the month of the survey period for each income group.

*Role of the funding source*

The funder of the study had no role in study design, data collection, data analysis, data interpretation, or writing of the report.

**Results**

The sample included 73,638 households in 11 low-income countries, 16 lower middle-income countries and 12 upper middle-income countries; 7 countries from East Asia and the Pacific, 13 from Latin America and the Caribbean, 3 from the Middle East and North Africa, and 16 from Sub-Saharan Africa. (Table 1).

[Insert Table 1 here]

In the pooled sample, 18·8% (95% CI 17·8%–19·8%) of households reported not being able to access health care when needed (Table 2; Figure 1). This proportion did not vary substantially by country income group: 17·8% (16·3%–19·3%) of households in low-income countries reported not being able to access health care when needed, compared with 17·8% (16·3%–19·4%) in lower middle-income countries, and 20·8% (18·9%–22·7%) in upper-middle income countries. There was greater variation in foregone among regions: 9·1% (7·8%–10·3%) of households in countries in East Asia and Pacific reported not being able to access health care when needed compared with 17·4% (16·0%–18·9%) in Sub-Saharan Africa; 22·0% (20·1%–23·9%) in Latin America and the Caribbean; and 36·8% (32·6%–41·1%) in the Middle Eastern and North Africa. The differences were statistically significant.

[Insert Table 2 here]

Among the households foregoing health care when needed, financial barriers, including lack of money for medical care or lack of transportation, were the most-commonly reported reason, cited by 31·4% (28·6%–34·3%) of households in the pooled sample (Figure 2). 25·4% (22·6%–28·2%) of household cited reasons related to lockdown measures or fear of contracting COVID-19 when seeking care, and 21·0% (18·4%–23·6%) cited reasons related to the supply of health services: health facilities being full or closed, or insufficient staff or supplies. 22·3% (19·6%–25·0%) of respondents cited other reasons for not accessing care, including not being able to get an appointment (common in Latin American middle-income countries) and other non-specified reasons.

The reasons for foregoing care varied substantially by country income group. In low-income countries, 65·6% (59·9%–71·2%) of households reported foregoing care due to financial constraints. This proportion was smaller in lower middle-income countries (30·6%[26·2%–35·1%]) and even smaller in upper middle-income countries (17·4%[13·1%–21·6%]). The reverse was true for fear of COVID-19 and lockdown measures. While 30·4% (25·9%–34·8%) of households in lower middle-income and 27·2% (22·5%–31·8%) in upper middle-income countries reported not being able to access services because of lockdowns or fear of COVID-19, only 8·0% (4·7%–11·3%) of households in low-income countries did so. The difference between the proportions of households reporting financial and COVID-19 related barriers to care were statistically significant. The proportions of households reporting being unable to access health services due to supply-side barriers (lack of personnel or facility closure) among low-, lower middle-, and upper middle-income countries were similar (19·1% [14·2%–24·0%], 20·9%[16·7%–25·1%], and 21·9%[17·7%–26·1%], respectively) and not statistically significant.

[Insert Figure 2 here]

Financial reasons for foregoing care were reported by 51·8% (46·6%–57·1%) of respondents from Sub-Saharan Africa, 43·6% (35·9%–51·2%) from East Asia and the Pacific, 27·8% (21·7%–34·0%) from the Middle East and North Africa, and 12·4% (8·6%–16·2%) from Latin America. COVID-19-related reasons were reported by 33·1% (25·6%–40·7%) of households from East Asia and the Pacific, 31·9% (27·3%–36·6%) from Latin America and the Caribbean, 25·7% (19·0%–32·5%) from Middle East and North Africa, and 16·7% (12·5%–20·9%) from Sub-Saharan Africa. Finally, supply-side barriers were reported by 28·8% (21·8%–35·7%) of households from the Middle East and North Africa, 24·6% (20·5%–28·7%) of households from Latin America, 16·9% (12·6%–21·2%) of households from Sub-Saharan Africa, and 8·9% (5·1%–12·8%) of households from East Asia and the Pacific. Each type of reason for each region was statistically different from the reference region - Sub-Saharan Africa.

[Insert Figure 3 here]

In Figure 3 we present population-weighted average total COVID-19 cases per million and lockdown stringency index by income group for the countries in our sample. We find on average the total number of COVID-19 cases per million in upper middle-income countries is 438, higher than the 148 cases per million and 17 cases per million of lower middle- and low-income countries respectively. We find the average lockdown stringency index was 81 in upper middle-income countries as compared to 78 in lower middle- and 75 low-income countries.

## Discussion

As of its writing, this is the first multi-country study to quantitatively assess the prevalence of foregone health care and reasons why households forego care during the COVID-19 pandemic in LMICs. The concept of foregone care focuses on conditions under which people chose not to, or are not able to, use health services despite perceiving a need for those services. As such, foregone care is distinct from service (non)utilization, which also depends on explicit need for services and on service availability. Therefore, the analyses presented above should not be interpreted as capturing fully disruptions in service utilization during the pandemic.

We report two key findings. First, in the early months of the pandemic a substantial proportion of households, 18·8% across all countries in the sample, reported not being able to access health care services they needed. Second, the reported barriers to accessing health care were different in different country income groups.

The proportion of households reporting foregone care due to lockdown measures or fear of contracting COVID-19 was the highest in the upper middle-income countries, lower in lower middle-income countries, and the lowest in low-income countries. Data from the US from March-July 2020 showing the highest proportion of adult respondents reporting foregoing care because of the fear of contracting COVID-19 (29%) and only 7% because of financial barriers, is consistent with the patterns we find for upper middle-income countries.(7) The findings are also consistent with the epidemiological profile of the pandemic, with higher relative mortality and morbidity recorded in richer countries (Figure 3, Panel A) and with the severity of the lockdown measures, which was higher during that time period in richer countries (Figure 3, Panel B). In contrast, the proportion of households reporting financial reasons for not being able to access health services was the highest in low-income countries, and lower in lower-middle income and upper-middle income countries.

Together, these findings imply that barriers to accessing care experienced during the pandemic will likely reduce in the short term in richer countries but will likely persist and perhaps event deepen in poorer countries. First, the key reasons behind foregone care in richer countries – fear of contracting COVID-19 and lockdowns – will likely substantially diminish with the rollout of COVID-19 vaccines and will do so sooner than in poorer countries. While richer countries have been able to vaccinate large proportion of their populations, the introduction of vaccines has been substantially slower in poorer countries and will likely continue to be so in the near future.

At the same time, financial barriers – the main reason for foregoing care in poorer countries – will will likely persist longer than those related to lockdowns and the fear of COVID-19. In 2020, the global economy is estimated to have contracted by 3·3%, reducing household incomes and pushing 95 million people into poverty.(15) Although positive economic growth at the global level is expected to return in 2021, the recovery is expected to be uneven. Based on the IMF projection, growth in advanced economies in 2021 will reach about 5·1%, but only 3·4% in Sub-Saharan Africa and medium-term economic losses are projected to be more pronounced in LMICs.(15) The global recession due to the pandemic has put an unprecedented strain on government budgets creating risks of diminishing government health expenditure and reducing households' capacity to cover out of pocket health care expenses.(16) The latter is of particular concern for lower income countries, where health care is financed to a much larger extent through out-of-pocket expenditure. A slower pace of recovery in lower income countries means that those risks will persist there longer and that the disparity between richer and poorer countries in financial barriers to accessing care will likely increase. Evidence from the global financial crisis of 2007-2009, confirms this risk. In the US and in Europe, the prevalence of foregone care increased during the recession and remained high during the immediate recovery period.(17, 18)

It is likely that a substantial proportion of households in LMICs were foregoing health care due to financial and other reasons before the pandemic. However, it is difficult to compare our estimates with pre-pandemic figures. Many of the face-to-face surveys from which the phone surveys samples we make use of were drawn did not collect information on foregone care. Those that did, used different methodological approaches (e.g. collected data in person from all household members, compared to the phone surveys collecting inform about the households from a single respondent) which prevents a direct comparison. The difference in methodology means observed differences in foregone care prevalence may be attributable to both the broader impact of the COVID-19 crisis or to the particulars of the measurement approach and disentangling the contribution of these is not possible.

In general, in contrast to robust estimates of catastrophic and impoverishing health expenditure,(19) the literature on foregone care in LMICs is remarkably limited. Although many studies have examined foregone care in high-income countries, there are no published analyses of pooled cross-regional and cross-country data from LMICs. Murphy and colleagues provided estimates of foregoing medications for non-communicable diseases due to financial reasons in 18 high-, middle-, and low-income countries.(20) Although those estimates are not directly comparable with our study, they show a similar pattern: the proportion of households reporting foregoing medication for financial reasons is higher in low-income countries and gradually declines in the higher income groups. A study synthesizing data from 20 African countries collected in 2008 and 2009 showed that between 35% (in Ghana) and 80% (in Zimbabwe) household went at least once in the 12 months preceding the data collection without either health services or medicines they needed.(21) Those estimates are substantially higher than the ones we report but they use a different recall period (12 months) and combine health services and medicine purchases. Three other published single-country studies from Africa (Ethiopia, Kenya, and Liberia) estimated the prevalence of foregone care using different methodological approaches.(22-24) A study from 27 countries in the Americas using data spanning 2000-2018 estimated the average prevalence of foregone care to be 29·3% for any health services and 16·7% for care for child illnesses.(25) However, foregone care was defined as not seeking care or seeking care that was not deemed to be appropriate. A study using International Social Survey Program (ISSP) data collected in 2011-2013 in 23 countries included estimates of foregone care in two upper middle-income countries: South Africa and Turkey (13·5% and 28·0%, respectively).(26) Finally, two published studies have estimated foregone care in China – one among older adults with chronic diseases and another among cancer survivors.(27, 28)

This paper focuses on country-level analysis, comparing differences in foregone care across different country income groups and regions of the world, to emphasize the heterogeneity in the reasons behind foregone care and to call attention to the need of potentially different policy responses during the recovery in poorer and richer countries. Future analyses will explore within country differences, and the extent to which different household-level characteristics (e.g. household income) affect the likelihood of households' foregoing care they need.

One potential limitation of this study is that it is based on phone survey data which exclude respondents who did not have access to a phone. This method of data collection was necessary to collect information quickly during the COVID-19 pandemic, while respecting local movement restrictions and minimizing the risk of COVID transmission. Given that phone posession may be non-random, this might have created a risk that the results are representative only of the population with access to phone. However, in the countries in this sample, access to mobile phones was generally high –the population weighted average mobile cellular subscriptions was 99·8 per 100 people in 2019.(29) Furthermore, sampling weights were used in the analysis to correct for the biases resulting from non-random access to phones. A recent analysis using the same phone survey samples we make use of from 4

African countries demonstrated that the weighting procedures successfully minimized the selection bias in the phone surveys for a wide range of indicators.(30)

Another potential limitation is that the estimates are based on self-reports. This approach is widely used in foregone care studies, but poses a risk of recall, response, and other types of bias for which alternatives have been proposed.(22, 24, 31) Mebratie and colleagues used clinical vignettes to assess whether households in Ethiopia used appropriate health services; Gabani and Guiness measured foregone care by estimating the proportion of household who faced a health shock but reported only very small expenditure which was deemed insufficient to obtain the necessary care.(22, 24) However, collecting data using such methods is more challenging, requires judgement calls from researchers, and, to date, there has been no empirical analysis of the performance of such measures vis a vis self-reports.

Finally, the data were collected from one respondent per household reporting for the entire household, rather than from each household member individually. It is possible that the respondent (usually the household head) may not have accurately recalled whether a household member needed care, whether they were not able to access it, and why. It is unclear how this could affect the reported reasons for foregoing care. It seems more likely that this source of bias would result in under-estimation of the prevalence of foregone care if the respondent did not recall or was not aware of household members needing and/or not being able to access care (for example, a male head of household might not have been aware of female household members foregoing reproductive health services). Therefore, the estimates presented here could be considered a likely lower-bound of foregone care.

In sum, the key implication of our paper is that the COVID-19 pandemic has exacerbated old, well-entrenched risks for foregoing health care in LMICs and created new ones. With the roll-out of national vaccination programs, the fear of contracting COVID-19 is expected to diminish, and lockdown measures are already being lifted in countries that have achieved high vaccination coverage. Economic recovery, however, will take longer and current projections suggest that income inequality between countries will increase in the aftermath of the pandemic. Our analyses suggest that so may disparities in access to health care. Therefore, to protect progress toward universal health coverage achieved to date in LMICs, in addition to COVID-19 vaccination, policy actions at the national and global level that ensure financial access to essential health services need to be a critical part of the recovery.

**Figure 1: Prevalence of foregone care**

ACCEPTED MA

**Figure 2: Prevalence of reasons for foregoing care**

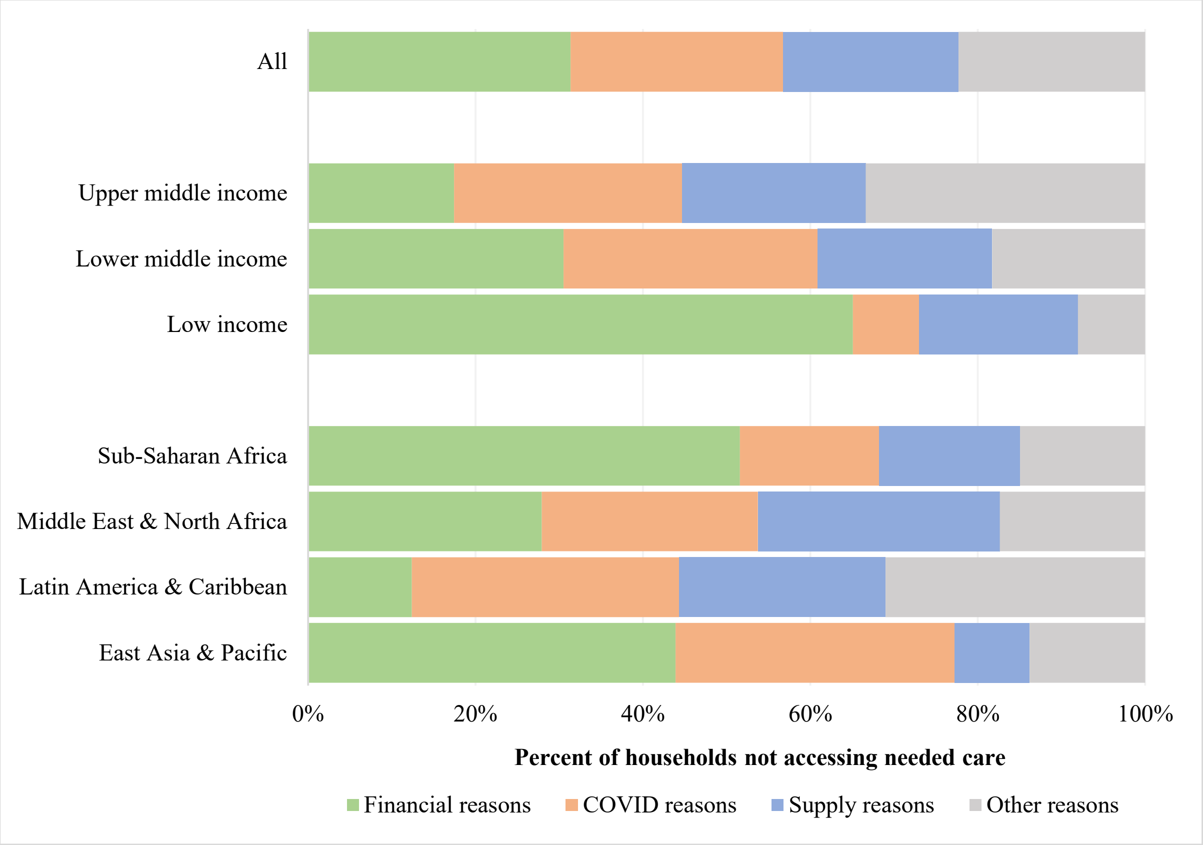

ACCEPTED M

**Figure 3: Prevalence of foregone care and country COVID-19 case burdens and policy responses**

***Panel A: Total COVID-19 Cases per million population during month of survey in 39 countries***

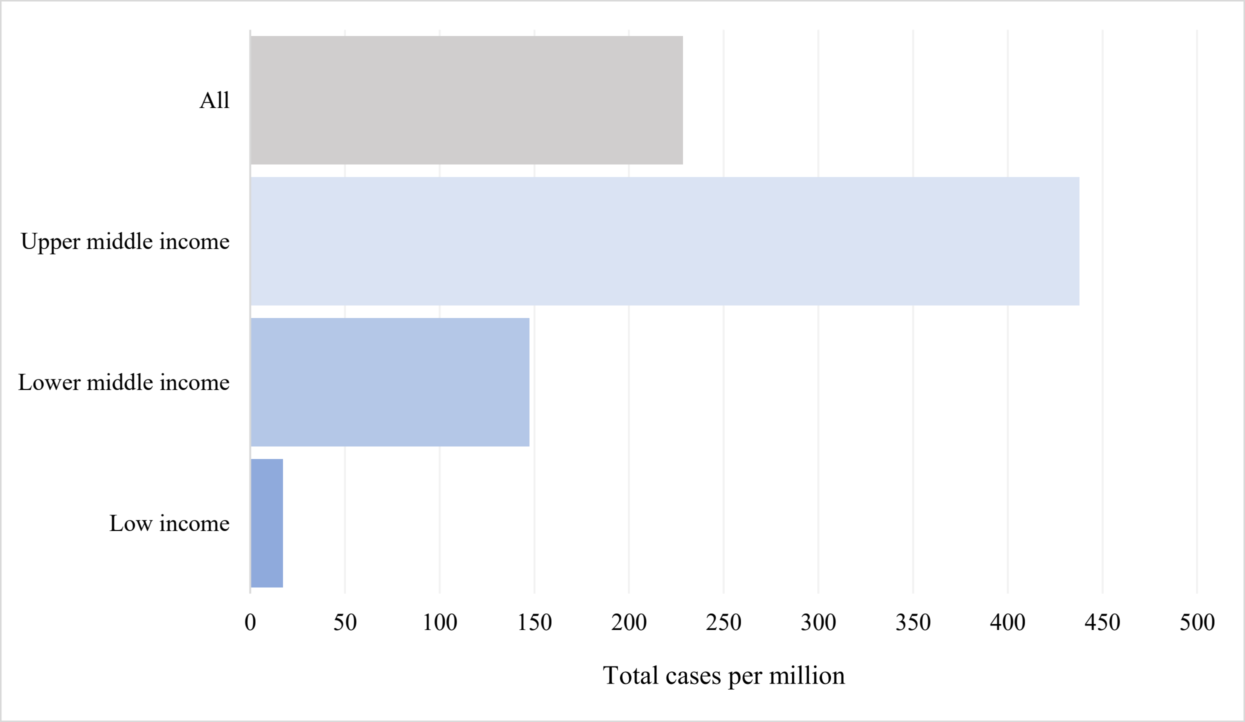

***Panel B: Oxford Stringency Index during month of survey in 39 countries***

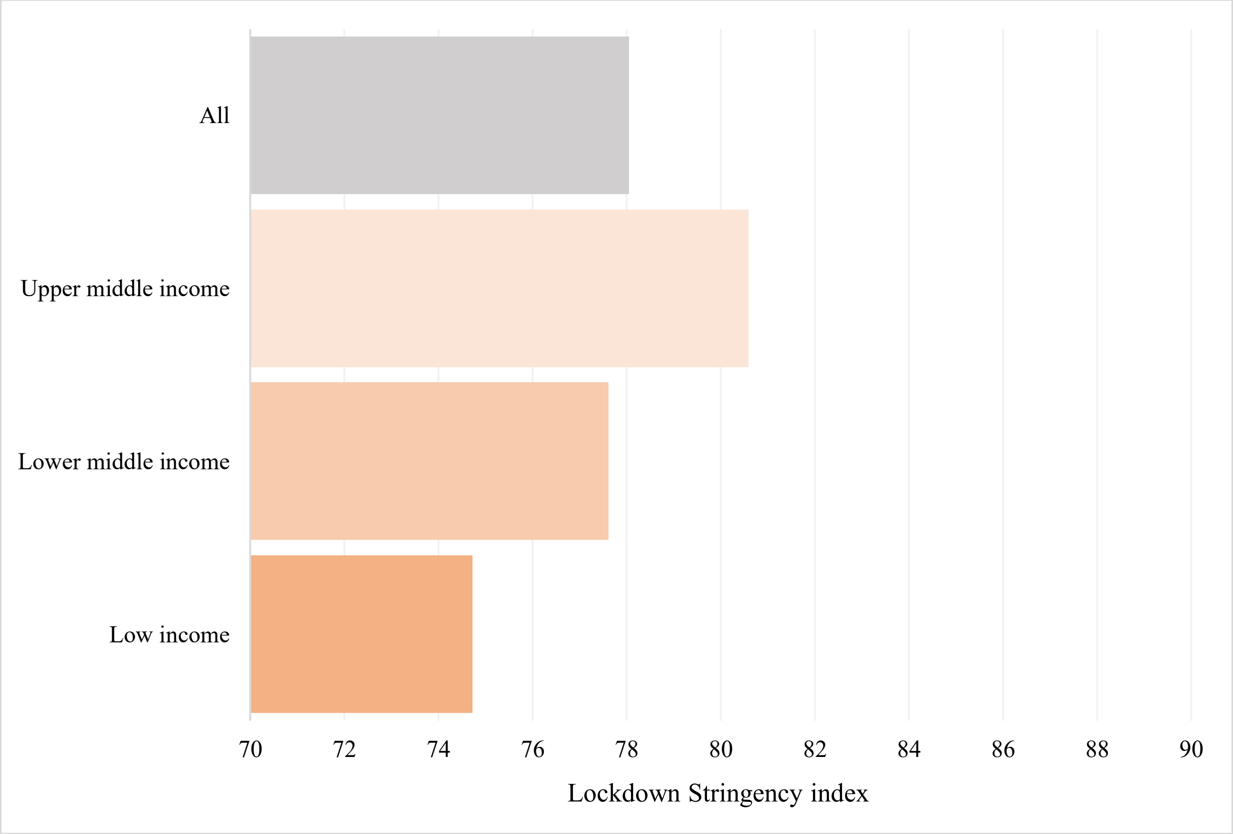

Table 1: Sample characteristics

| | | (1) | (2) |
|---|---|---|---|
| Characteristic | | Household observations | Country surveys |
| Income group | Low-income | 19,938 (27.1%) | 11 (28%) |
| | Lower middle-income | 37,762 (51.3%) | 16 (41%) |
| | Upper middle-income | 15,938 (21.6%) | 12 (31%) |
| Region | East Asia & Pacific | 27,428 (37.2%) | 7 (18%) |
| | Latin America & Caribbean | 12,479 (16.9%) | 13 (33%) |
| | Middle East & North Africa | 4,139 (5.6%) | 3 (8%) |
| | Sub-Saharan Africa | 29,592 (40.2%) | 16 (41%) |
| Month | May | 11,911 (16.2%) | 9 (23%) |
| | June | 43,861 (59.6%) | 25 (64%) |
| | July | 6,797 (9.2%) | 3 (8%) |
| | August | 11,069 (15.0%) | 2 (5%) |
| N | | 73,638 | 39 |

**Note:** Data are from World Bank's High Frequency Phone Surveys fielded between May and August of 2020.

**Table 2:** Prevalence of foregone care and reasons for foregoing care

| | (1) | (2) | (3) | (4) | (5) |
|---|---|---|---|---|---|
| | Prevalence of foregone care | Prevalence of reasons for foregoing care | | | |
| | | (i) Financial reasons | (ii) COVID reasons | (iii) Supply reasons | (iv) Other reasons |
| | *Percentage of households* | *Percentage of households not accessing care* | *Percentage of households not accessing care* | *Percentage of households not accessing care* | *Percentage of households not accessing care* |
| **All countries** | | | | | |
| All countries | 18.8 (17.8 - 19.8) | 31.4 (28.6 - 34.3) | 25.4 (22.6 - 28.2) | 21.0 (18.4 - 23.6) | 22.3 (19.6 - 25.0) |
| **Income group** | | | | | |
| Low Income | 17.8 (16.3 - 19.3) | 65.6 (59.9 - 71.2) | 8.0 (4.7 - 11.3) | 19.1 (14.2 - 24.0) | 8.1 (5.1 - 11.0) |
| Lower middle-income | 17.8( 16.3 - 19.4) | 30.6*** (26.2 - 35.1) | 30.4*** (25.9 - 34.8) | 20.9 (16.7 - 25.1) | 18.3*** (14.3 - 22.3) |
| Upper middle-income | 20.8** (18.9 - 22.7) | 17.4*** (13.1 - 21.6) | 27.2*** (22.5 - 31.8) | 21.9 (17.7 - 26.1) | 33.3*** (28.4 - 38.2) |
| **Region** | | | | | |
| East Asia & Pacific | 9.1*** (7.8 - 10.3) | 43.6* (35.9 - 51.2) | 33.1*** (25.6 - 40.7) | 8.9*** (5.1 - 12.8) | 13.7 (8.3 - 19.0) |
| Latin America & Caribbean | 22.0*** (20.1 - 23.9) | 12.4***( 8.6 - 16.2) | 31.9*** (27.3 - 36.6) | 24.6** (20.5 - 28.7) | 31.0*** (26.4 - 35.7) |
| Middle East & North Africa | 36.8*** (32.6 - 41.1) | 27.8*** (21.7 - 34.0) | 25.7** (19.0 - 32.5) | 28.8*** (21.8 - 35.7) | 17.3 (12.4 - 22.2) |
| Sub-Saharan Africa | 17.4 (16.0 - 18.9) | 51.8 (46.6 - 57.1) | 16.7 (12.5 - 20.9) | 16.9 (12.6 - 21.2) | 15.0 (11.0 - 18.9) |

**Notes:** Data are from World Bank's High Frequency Phone Surveys fielded between May and August of 2020. Sample is restricted to households reporting indicating some healthcare need during survey's recall period. Prevalence of foregone care is the proportion of households who report needing care but not accessing needed care. Financial reasons are: lack of money and lack of transportation. COVID reasons are: fear of COVID, and movement restrictions. Supply reasons are: lack of medical personnel, lack of supplies/medication or facility closed/full.Large sample z-tests of proportion equality between (i) low income country households and others, and (ii) Sub-Saharan African country households and others respectively, are indicated by stars, with p <0.01 ***, p<0.05 **, and p <0.10 *

**Annex Table 1: Surveys included in sample**

| Country | Month | Income group | Region |
|---|---|---|---|
| Argentina | June | Upper middle income | Latin America & Caribbean |
| Burkina Faso | June | Low income | Sub-Saharan Africa |
| Bolivia | June | Lower middle income | Latin America & Caribbean |
| Congo, Dem. Rep. | June | Low income | Sub-Saharan Africa |
| Colombia | June | Upper middle income | Latin America & Caribbean |
| Costa Rica | May | Upper middle income | Latin America & Caribbean |
| Djibouti | July | Lower middle income | Middle East & North Africa |
| Dominican Republic | May | Upper middle income | Latin America & Caribbean |
| Ecuador | June | Upper middle income | Latin America & Caribbean |
| Ethiopia | June | Low income | Sub-Saharan Africa |
| Ghana | June | Lower middle income | Sub-Saharan Africa |
| Guatemala | May | Upper middle income | Latin America & Caribbean |
| Honduras | June | Lower middle income | Latin America & Caribbean |
| Indonesia | May | Upper middle income | East Asia & Pacific |
| Iraq | August | Upper middle income | Middle East & North Africa |
| Cambodia | May | Lower middle income | East Asia & Pacific |
| Lao PDR | July | Lower middle income | East Asia & Pacific |
| St. Lucia | May | Upper middle income | Latin America & Caribbean |
| Madagascar | June | Low income | Sub-Saharan Africa |
| Mexico | June | Upper middle income | Latin America & Caribbean |
| Mali | June | Low income | Sub-Saharan Africa |
| Mongolia | May | Lower middle income | East Asia & Pacific |
| Mozambique | June | Low income | Sub-Saharan Africa |
| Malawi | June | Low income | Sub-Saharan Africa |
| Nigeria | June | Lower middle income | Sub-Saharan Africa |
| Peru | June | Upper middle income | Latin America & Caribbean |
| Philippines | August | Lower middle income | East Asia & Pacific |
| Papua New Guinea | June | Lower middle income | East Asia & Pacific |
| Paraguay | June | Upper middle income | Latin America & Caribbean |
| Senegal | May | Lower middle income | Sub-Saharan Africa |
| El Salvador | June | Lower middle income | Latin America & Caribbean |
| Somalia | July | Low income | Sub-Saharan Africa |
| South Sudan | June | Low income | Sub-Saharan Africa |
| Chad | June | Low income | Sub-Saharan Africa |
| Tunisia | May | Lower middle income | Middle East & North Africa |
| Uganda | June | Low income | Sub-Saharan Africa |
| Vietnam | June | Lower middle income | East Asia & Pacific |
| Zambia | June | Lower middle income | Sub-Saharan Africa |
| Zimbabwe | June | Lower middle income | Sub-Saharan Africa |

**Annex Table 2: Prevalence of specific reasons for foregoing care**

| | Lack of money | Lack of transportation | Afraid/ concerned about catching COVID | Movement restrictions (stay–at–home orders) | No medical personnel available | Facility was full | Facility was closed | Hospital/clinic did not have enough supplies or tests | Other |
|---|---|---|---|---|---|---|---|---|---|
| | Percentage of households not accessing care | Percentage of households not accessing care | Percentage of households not accessing care | Percentage of households not accessing care | Percentage of households not accessing care | Percentage of households not accessing care | Percentage of households not accessing care | Percentage of households not accessing care | Percentage of households not accessing care |
| **All countries** | | | | | | | | | |
| All Countries | 25·6(23·1–28·2) | 6·1(4·5–7·8) | 15·4(13·2–17·6) | 10·0(8·0–12·1) | 18·7(16·2–21·3) | 1·7(1·0–2·4) | 0·3(0·1–0·4) | 0·4(0·1–0·7) | 22·3(19·6–25·0) |
| **Country income group** | | | | | | | | | |
| Low Income | 56·9(51·2–62·7) | 9·5(6·4–12·7) | 5·1(2·6–7·6) | 2·9(0·6–5·2) | 13·6(9·3–17·9) | 4·1(1·5–6·7) | 0·4(–0·1–0·9) | 1·6(0·1–3·1) | 8·1(5·1–11·0) |
| Lower middle–income | 24·5***(20·6–28·3) | 6·6(3·6– 9·6) | 18·2***(14·5–21·8) | 12·2***(8·9–15·5) | 19·2*(15·1–23·3) | 1·8(0·8–2·9) | 0·0(..) | 0·0(..) | 18·3***(14·3–22·3) |
| Upper middle–income | 13·2***(9·3–17·2) | 4·1***(2·1–6·2) | 16·6***(12·8–20·3) | 10·6***(7·1–14·1) | 20·5**(16·4–24·6) | 0·6*(0·0–1·1) | 0·5(0·1–1·0) | 0·3(–0·0–0·6) | 33·3***(28·4–38·2) |
| **Region** | | | | | | | | | |
| East Asia & Pacific | 41·0(33·5–48·6) | 2·5***(0·4–4·7) | 29·4(21·9–36·9) | 3·8(1·4–6·1) | 3·8***(1·2–6·4) | 4·0(1·6–6·4) | 0·0(..) | 1·7(–0·2–3·6) | 13·7(8·3–19·0) |
| Latin America & Caribbean | 6·4***(3·1–9·7) | 6·0(3·8–8·2) | 19·8(15·9–23·7) | 12·1**(8·8–15·5) | 24·6***(20·5–28·7) | 0·0***(0·0–0·0) | 0·0(..) | 0·0(..) | 31·0***(26·4–35·7) |
| Middle East & North Africa | 27·8***(21·7–34·0) | 0·0(..) | 5·9**(3·0–8·9) | 19·8***(13·3–26·3) | 24·6***(17·9–31·4) | 2·0(–0·1–4·1) | 2·1(0·5–3·7) | 0·0(..) | 17·3(12·4–22·2) |
| Sub–Saharan Africa | 44·3(39·3–49·3) | 8·4(5·0–11·7) | 10·4(7·2–13·6) | 6·3(3·2–9·4) | 13·1(9·1–17·2) | 3·2(1·6–4·9) | 0·2(–0·1–0·4) | 0·7(0·0–1·3) | 15·0(11·0–18·9) |

**Notes:** Data are from World Bank's High Frequency Phone Surveys fielded between May and August of 2020. Sample is restricted to households reporting indicating some healthcare need during survey's recall period. Prevalence of foregone care is the proportion of households who report needing care but not accessing needed care. Financial reasons are: lack of money and lack of transportation. COVID reasons are: fear of COVID, and movement restrictions. Supply reasons are: lack of medical personnel, lack of supplies/medication or facility closed/full. Totals of columns (1) – (9) may not add up to 100% exactly due to rounding and as 3 surveys allowed respondents to indicate more than one reason for not accessing care. Large sample z-tests of proportion equality between (i) low income country households and others, and (ii) Sub-Saharan African country households and others respectively, are indicated by stars, with p <0.01 ***, p<0.05 **, and p <0.10

**Weighting-adjustment for pooled estimates:**

As we are pooling samples from different countries, there is a risk that smaller countries may be over-represented in final samples if weights are not adjusted. To address this concern, we adopt a post-stratification approach – scaling the original sampling weights for each country's survey by the inverse of that country's share of the country group population (See *§8.5.2 Poststratification* in Lohr (2019)[1]).

Specifically, we first construct a post-stratification weight for each country $c$ in country group $g$ as:

$$w_{ps,g,c} = \frac{\sum_{j \in g} Population_j}{Population_c}$$

This is then used to scale each country's sampling weights to produce a final weight. For household $h$, in country $c$ in country group $g$, their original sampling weight, $w_{sample,g,c,h}$, is multiplied by the post stratification weight as follows:

$$w_{final,g,c,i} = w_{sample,g,c,h} . w_{ps,g,c}$$

[1] Lohr SL. Sampling: design and analysis. Chapman and Hall/CRC; 2019.
