# Foregone healthcare during the COVID-19 pandemic: early survey estimates from 39 low- and middle-income countries

**DOI:** 10.1093/heapol/czac024

**Published:** 2022-03-11

**Authors:** Jakub Jan Kakietek, Julia Dayton Eberwein, Nicholas Stacey, David Newhouse, Nobuo Yoshida

**Affiliations:** The World Bank, 1818 H. Street, NW, Washington, DC 20433, USA; The World Bank, 1818 H. Street, NW, Washington, DC 20433, USA; Department of Health Policy, London School of Economics and Political Science, 6 Portugal Street, London WC2A 2HD, UK; The World Bank, 1818 H. Street, NW, Washington, DC 20433, USA; The World Bank, 1818 H. Street, NW, Washington, DC 20433, USA

**Keywords:** Foregone healthcare, COVID-19, LMICs

## Abstract

In addition to the direct health effects of the Coronavirus disease (COVID-19) pandemic, the pandemic has increased the risks of foregone non-COVID-19 healthcare. Likely, these risks are greatest in low- and middle-income countries (LMICs), where health systems are less resilient and economies more fragile. However, there are no published studies on the prevalence of foregone healthcare in LMICs during the pandemic. We used pooled data from phone surveys conducted between April and August 2020, covering 73 638 households in 39 LMICs. We estimated the prevalence of foregone care and the relative importance of various reported reasons for foregoing care, disaggregated by country income group and region. In the sample, 18.8% (95% CI 17.8–19.8%) of households reported not being able to access healthcare when needed. Financial barriers were the most-commonly self-reported reason for foregoing care, cited by 31.4% (28.6–34.3%) of households. More households in wealthier countries reported foregoing care for reasons related to COVID-19 [27.2% (22.5–31.8%) in upper-middle-income countries compared to 8.0% (4.7–11.3%) in low-income countries]; more households in poorer countries reported foregoing care due to financial reasons [65.6% (59.9–71.2%)] compared to 17.4% (13.1–21.6%) in upper-middle-income countries. A substantial proportion of households in LMICs had to forgo healthcare in the early months of the pandemic. While in richer countries this was largely due to fear of contracting COVID-19 or lockdowns, in poorer countries foregone care was due to financial constraints.

Key messages

- A significant proportion of households in LMICs had to forego needed healthcare during the COVID-19 pandemic.
- While in richer countries this was largely reported as due to fear of contracting COVID-19 or lockdowns, in poorer countries foregone care was reported as due to financial constraints.

## Introduction

As of May 2021, there have been over 2 million COVID-19 deaths and 93 million confirmed COVID-19 cases in low- and middle-income countries (LMICs)—about 56% of the total global burden of mortality and 54% of morbidity from the disease (Dong *et al.*, 2020). In addition to its direct impact, the pandemic has increased the risk of foregone non-COVID-19 care (WHO, 2020a). There are concerns that many individuals may have chosen not use health services because of fear of being exposed to COVID-19 at healthcare facilities, because of movement and other limitations imposed by national lockdowns and due to financial constraints resulting from the economic downturn. The pandemic may have also negatively affected the supply of health services by overwhelming health facilities with large numbers of COVID-19 patients, diverting the use of resource from essential health services to COVID-19 management and care, or disrupting supply chains for commodities and equipment. Those demand and supply risks are particularly elevated in LMICs, where health systems are less resilient and economies more fragile. During the 2014–15 Ebola outbreak, average healthcare utilization declined by 18%, with even larger declines for maternal and child health services (Brolin Ribacke *et al.*, 2016; Sochas *et al.*, 2017; Wilhelm and Helleringer 2019). Modelling studies have shown that foregone utilization of essential health services may lead to substantial increases in mortality and morbidity (Roberton *et al.*, 2020).

Emerging evidence from developed countries suggests that the utilization of some essential services declined during the COVID-19 pandemic, and recent studies have shown increased incidence of foregone care in the USA and the European Union (Santoli *et al.*, 2020; Anderson *et al.*, 2021; Ksinan Jiskrova *et al.*, 2021). However, despite greater vulnerability of households and fragility of the health systems, there are no published studies on the prevalence of foregone healthcare during the pandemic in LMICs. WHO pulse surveys of key respondents carried out in August 2020 and April 2021 presented data on perceptions of health sector government officials of the disruption in the provision of health services but provided no quantitative data on the prevalence of foregone care or the types of barriers to utilization service users face (WHO, 2020b). A systematic review of the literature found that maternal and foetal outcomes have worsened during the pandemic, but it included data from only two LMICs (Turkey and Nepal) (Chmielewska *et al.*, 2021). In contrast, a study of women’s need for and use of contraceptives in Burkina Faso, Democratic Republic of Congo, Kenya and Nigeria during the COVID-19 pandemic relative to the pre-COVID-19 period found mixed results: the need for contraception increased in Nigeria, and contraceptive use increased in Kenya and rural Burkina Faso (Wood *et al.*, 2021). None of the studies directly assessed whether individuals or households forewent the care they needed because of reasons directly related to COVID-19 or other reasons.

This study fills a critical evidence gap by providing estimates of foregone care during the early months of the pandemic in 39 LMICs, which account for 24.6% of the total population of LMICs, and 43.1% excluding India and China. Based on phone survey data from over 73 000 households and collected using a largely harmonized questionnaire and methods to assess foregone care, the data allow to produce estimates of the prevalence of foregone care and the prevalence of reasons for foregoing care across country income groups and regions. While aimed at improving the understanding of foregone care during the pandemic, the analyses also constitute an important contribution to the general literature on foregone care in LMICs.

## Methods

### Data

This study used pooled household survey data from 39 high-frequency phone-based surveys conducted between April and August 2020 as part of the World Bank’s initiative to monitor the socio-economic impact of COVID-19 (World Bank, 2020). Supplementary Annex Table S1 presents a list of countries and the month of survey data collection. The samples were drawn in three main ways. In 16 countries, the sample was drawn from a recent large in-person population survey, such as the Living Standard Measurement Survey. In another 16 cases, the samples were selected through random digit dialling. In another seven cases, the samples were drawn from a pre-existing list of phone numbers. The surveys were designed to be representative of the target group at the national level. To correct for the bias resulting from the non-random ownership of phones, in each survey, sampling weights were developed to adjust for the likelihood of the respondent household owning a phone and other household characteristics. Information was collected from one respondent per household, usually the household head, who respondent on behalf of the entire household.

### Outcome measures

A generic questionnaire served as the basis for each country’s survey, but in most cases some modifications were made to fit the local contexts. All surveys asked two questions related to the utilization of health services: (1) whether the respondent or any member of their household needed medical care during the recall period (usually, 30 days); (2) whether the respondent/household member was able to access the services they needed. A subset of 35 surveys asked a third question to respondents who could not access care: what was the main reason the respondent/household member could not access the services they needed. In most surveys, this last question was posed as an open-ended question, with the survey enumerator categorizing the answer using pre-identified categories. The categories included in most surveys were as follows: financial barriers—(1) lack of money; (2) lack of transportation; reasons directly related to COVID-19—(3) fear of contracting COVID-19; (4) movement restrictions/lockdown/stay-at-home orders; reasons related to the supply of health services—(5) no medical personnel available at health facility; (6) turned away because facility was full; (7) no medication/stockout; and (8) other (specify). Some surveys included additional categories (e.g. visiting a traditional healer). Those were combined with the ‘other’ category. For simplicity, in the analyses presented below, the reasons for forgoing care were grouped into four categories: (1) financial barriers (lack of money; lack of transportation); (2) lockdowns and fear of COVID; (3) reasons related to the supply of health services (no medical personnel available; facility was full) and (4) other reasons. The results disaggregated by each of the eight categories listed above are available in the Supplementary Annex Table S2. Consistent with the literature, households were considered to forego care when they reported that they needed care and that they could not access the care they needed. The prevalence of foregone care was calculated as the households who could not access care as the percentage of the households reporting needing care.

### Statistical analysis

The statistical analysis was descriptive in nature and focused on documenting the extent of reported foregone care, reasons for household foregoing care and differences in those indicators among countries from different regions and income groups. Data from the 39 surveys were pooled into a single data set. Point estimates and 95% confidence intervals are reported for the pooled sample and by country income group and global region. In addition, large sample two-sample tests of proportion equivalence were carried out to compare low-income countries with (1) lower-middle-income countries and (2) upper-middle-income countries, and Sub-Saharan African countries with (1) East-Asia and Pacific countries, (2) Latin American and Caribbean countries and (3) Middle East and North African countries. For pooled sample analysis, household sampling weights were adjusted by the country’s population as a proportion of the population of all countries included in the study. For income group and region estimates, sampling weights for observations of each country were adjusted by their country’s population as a proportion of the total population of the countries in the grouping, so that each country’s contribution to the average was proportional to its population (see Annex for further details).

### Contextual measures

To provide further context to the estimates of foregone care prevalence, we construct measures of COVID burden and lockdown stringency at the country income group level. These are the average of the number of total cases per million in the countries included in the income group [sourced from Dong *et al.* (2020)], weighted by country population. Similarly, we construct a population-weighted average of the lockdown stringency index of Hale *et al.* (2021) for the month of the survey period for each income group.

### Role of the funding source

The funder of the study had no role in study design, data collection, data analysis, data interpretation or writing of the report.

## Results

The sample included 73 638 households in 11 low-income countries, 16 lower-middle-income countries and 12 upper-middle-income countries; 7 countries from East Asia and the Pacific, 13 from Latin America and the Caribbean, 3 from the Middle East and North Africa and 16 from Sub-Saharan Africa (Table 1).

**Figure 1.**
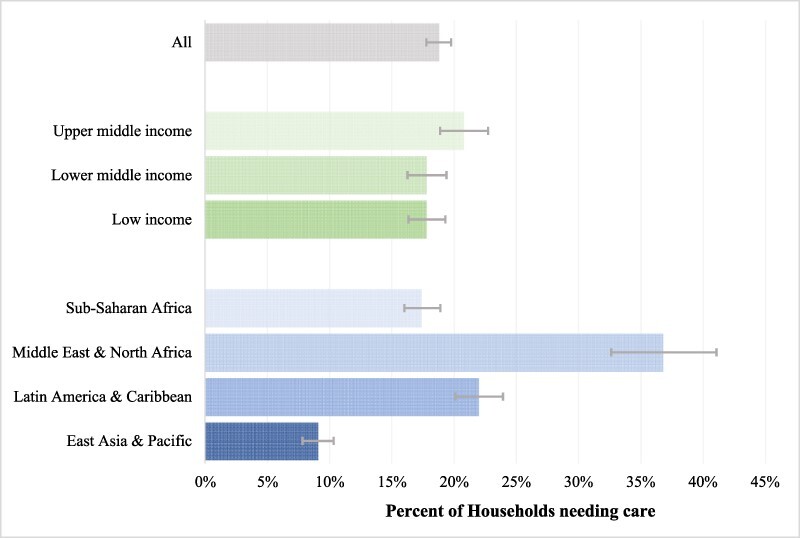
Prevalence of foregone care

In the pooled sample, 18.8% (95% CI 17.8–19.8%) of households reported not being able to access healthcare when needed (Table 2; Figure 1). This proportion did not vary substantially by country income group: 17.8% (16.3–19.3%) of households in low-income countries reported not being able to access healthcare when needed, compared with 17.8% (16.3–19.4%) in lower-middle-income countries and 20.8% (18.9–22.7%) in upper-middle-income countries. There was greater variation in foregone among regions: 9.1% (7.8–10.3%) of households in countries in East Asia and Pacific reported not being able to access healthcare when needed compared with 17.4% (16.0–18.9%) in Sub-Saharan Africa; 22.0% (20.1–23.9%) in Latin America and the Caribbean; and 36.8% (32.6–41.1%) in the Middle Eastern and North Africa. The differences were statistically significant.

**Table 1.** Sample characteristics

		(1)	(2)
Characteristics		Household observations	Country surveys
Income group	Low-income	19 938 (27.1%)	11 (28%)
	Lower-middle-income	37 762 (51.3%)	16 (41%)
	Upper-middle-income	15 938 (21.6%)	12 (31%)
Region	East Asia and Pacific	27 428 (37.2%)	7 (18%)
	Latin America and Caribbean	12 479 (16.9%)	13 (33%)
	Middle East and North Africa	4139 (5.6%)	3 (8%)
	Sub-Saharan Africa	29 592 (40.2%)	16 (41%)
Month	May	11 911 (16.2%)	9 (23%)
	June	43 861 (59.6%)	25 (64%)
	July	6797 (9.2%)	3 (8%)
	August	11 069 (15.0%)	2 (5%)
*N*		73 638	39

Note: Data are from World Bank’s High-Frequency Phone Surveys fielded between May and August of 2020.

**Table 2.** Prevalence of foregone care and reasons for foregoing care

	(1)	(2)	(3)	(4)	(5)
	Prevalence of foregone care	Prevalence of reasons for foregoing care			
		(i) Financial reasons	(ii) COVID reasons	(iii) Supply reasons	(iv) Other reasons
	***Percentage of households***	***Percentage of households not accessing care***	***Percentage of households not accessing care***	***Percentage of households not accessing care***	***Percentage of households not accessing care***
All countries					
All countries	18.8 (17.8–19.8)	31.4 (28.6–34.3)	25.4 (22.6–28.2)	21.0 (18.4–23.6)	22.3 (19.6–25.0)
Income group					
Low income	17.8 (16.3–19.3)	65.6 (59.9–71.2)	8.0 (4.7–11.3)	19.1 (14.2–24.0)	8.1 (5.1–11.0)
Lower-middle-income	17.8(16.3–19.4)	30.6*** (26.2–35.1)	30.4*** (25.9–34.8)	20.9 (16.7–25.1)	18.3*** (14.3–22.3)
Upper-middle-income	20.8** (18.9–22.7)	17.4*** (13.1–21.6)	27.2*** (22.5–31.8)	21.9 (17.7–26.1)	33.3*** (28.4–38.2)
Region					
East Asia and Pacific	9.1*** (7.8–10.3)	43.6* (35.9–51.2)	33.1*** (25.6–40.7)	8.9*** (5.1–12.8)	13.7 (8.3–19.0)
Latin America and Caribbean	22.0*** (20.1–23.9)	12.4***(8.6–16.2)	31.9*** (27.3–36.6)	24.6** (20.5–28.7)	31.0*** (26.4–35.7)
Middle East and North Africa	36.8*** (32.6–41.1)	27.8*** (21.7–34.0)	25.7** (19.0–32.5)	28.8*** (21.8–35.7)	17.3 (12.4–22.2)
Sub-Saharan Africa	17.4 (16.0–18.9)	51.8 (46.6–57.1)	16.7 (12.5–20.9)	16.9 (12.6–21.2)	15.0 (11.0–18.9)

Notes: Data are from World Bank’s High-Frequency Phone Surveys fielded between May and August of 2020. The sample is restricted to households reporting indicating some healthcare need during the survey’s recall period. The prevalence of foregone care is the proportion of households who report needing care but not accessing needed care. Financial reasons are lack of money and lack of transportation. COVID reasons are fear of COVID and movement restrictions. Supply reasons are the lack of medical personnel and the lack of supplies/medication or facility closed/full. Large sample *z*-tests of proportion equality between (i) low-income country households and others and (ii) Sub-Saharan African country households and others, respectively, are indicated by stars, with ***P < 0.01,

** *P* < 0.05, and

* *P* < 0.10.

**Figure 2.**
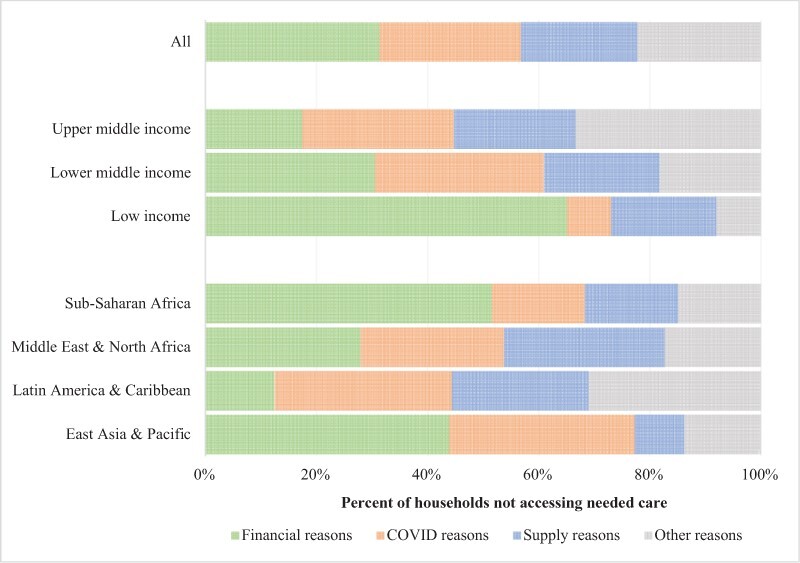
Prevalence of reasons for foregoing care

Among the households foregoing healthcare when needed, financial barriers, including lack of money for medical care or lack of transportation, were the most commonly reported reason, cited by 31.4% (28.6–34.3%) of households in the pooled sample (Figure 2). In total, 25.4% (22.6–28.2%) of household cited reasons related to lockdown measures or fear of contracting COVID-19 when seeking care, and 21.0% (18.4–23.6%) cited reasons related to the supply of health services: health facilities being full or closed, or insufficient staff or supplies. However, 22.3% (19.6–25.0%) of respondents cited other reasons for not accessing care, including not being able to get an appointment (common in Latin American middle-income countries) and other non-specified reasons.

The reasons for foregoing care varied substantially by country income group. In low-income countries, 65.6% (59.9–71.2%) of households reported foregoing care due to financial constraints. This proportion was smaller in lower-middle-income countries [30.6% (26.2–35.1%)] and even smaller in upper-middle-income countries [17.4% (13.1–21.6%)]. The reverse was true for fear of COVID-19 and lockdown measures. While 30.4% (25.9–34.8%) of households in lower-middle-income and 27.2% (22.5–31.8%) in upper-middle-income countries reported not being able to access services because of lockdowns or fear of COVID-19, only 8.0% (4.7–11.3%) of households in low-income countries did so. The difference between the proportions of households reporting financial and COVID-19-related barriers to care was statistically significant. The proportions of households reporting being unable to access health services due to supply-side barriers (lack of personnel or facility closure) among low-, lower-middle- and upper-middle-income countries were similar [19.1% (14.2–24.0%), 20.9% (16.7–25.1%) and 21.9% (17.7–26.1%), respectively] and not statistically significant.

Financial reasons for foregoing care were reported by 51.8% (46.6–57.1%) of respondents from Sub-Saharan Africa, 43.6% (35.9–51.2%) from East Asia and the Pacific, 27.8% (21.7–34.0%) from the Middle East and North Africa and 12.4% (8.6–16.2%) from Latin America. COVID-19-related reasons were reported by 33.1% (25.6–40.7%) of households from East Asia and the Pacific, 31.9% (27.3–36.6%) from Latin America and the Caribbean, 25.7% (19.0–32.5%) from Middle East and North Africa and 16.7% (12.5–20.9%) from Sub-Saharan Africa. Finally, supply-side barriers were reported by 28.8% (21.8–35.7%) of households from the Middle East and North Africa, 24.6% (20.5–28.7%) of households from Latin America, 16.9% (12.6–21.2%) of households from Sub-Saharan Africa and 8.9% (5.1–12.8%) of households from East Asia and the Pacific. Each type of reason for each region was statistically different from the reference region—Sub-Saharan Africa.

**Figure 3.**
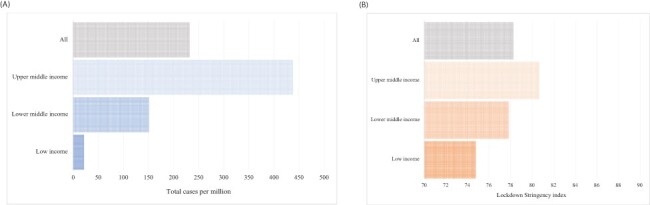
Prevalence of foregone care and country COVID-19 case burdens and policy responses. Panel A: Total COVID-19 cases per million population during month of survey in 39 countries. Panel B: Oxford Stringency Index during month of survey in 39 countries

In Figure 3, we present the population-weighted average total COVID-19 cases per million and lockdown stringency index by income group for the countries in our sample. We find on average the total number of COVID-19 cases per million in upper-middle-income countries is 438, higher than the 148 cases per million and 17 cases per million of lower-middle- and low-income countries, respectively. We find that the average lockdown stringency index was 81 in upper-middle-income countries as compared to 78 in lower-middle- and 75 low-income countries.

## Discussion

As of its writing, this was the first multi-country study to quantitatively assess the prevalence of foregone healthcare and reasons why households forego care during the COVID-19 pandemic in LMICs. The concept of foregone care focuses on conditions under which people chose not to, or are not able to, use health services despite perceiving a need for those services. As such, foregone care is distinct from service (non)utilization, which also depends on the explicit need for services and on service availability. Therefore, the analyses presented above should not be interpreted as capturing fully disruptions in service utilization during the pandemic.

We reported two key findings. First, in the early months of the pandemic a substantial proportion of households, 18.8% across all countries in the sample, reported not being able to access healthcare services they needed. Second, the reported barriers to accessing healthcare were different in different country income groups.

The proportion of households reporting foregone care due to lockdown measures or fear of contracting COVID-19 was the highest in the upper-middle-income countries, lower in lower-middle-income countries and the lowest in low-income countries. Data from the USA from March to July 2020 showing the highest proportion of adult respondents reporting foregoing care because of the fear of contracting COVID-19 (29%) and only 7% because of financial barriers are consistent with the patterns we find for upper-middle-income countries (Anderson *et al.*, 2021). The findings are also consistent with the epidemiological profile of the pandemic, with higher relative mortality and morbidity recorded in richer countries (Figure 3, panel A) and with the severity of the lockdown measures, which was higher during that time period in richer countries (Figure 3, panel B). In contrast, the proportion of households reporting financial reasons for not being able to access health services was the highest in low-income countries and lower in lower-middle-income and upper-middle-income countries.

Together, these findings imply that barriers to accessing care experienced during the pandemic will likely reduce in the short term in richer countries but will likely persist and perhaps event deepen in poorer countries. First, the key reasons behind foregone care in richer countries—fear of contracting COVID-19 and lockdowns—will likely substantially diminish with the roll-out of COVID-19 vaccines and will do so sooner than in poorer countries. While richer countries have been able to vaccinate large proportions of their populations, the introduction of vaccines has been substantially slower in poorer countries and will likely continue to be so in the near future.

At the same time, financial barriers—the main reason for foregoing care in poorer countries—will likely persist longer than those related to lockdowns and the fear of COVID-19. In 2020, the global economy is estimated to have contracted by 3.3%, reducing household incomes and pushing 95 million people into poverty (International Monetary Fund, 2021). Although positive economic growth at the global level is expected to return in 2021, the recovery is expected to be uneven. Based on the IMF projection, growth in advanced economies in 2021 will reach about 5.1%, but only 3.4% in Sub-Saharan Africa and medium-term economic losses are projected to be more pronounced in LMICs (International Monetary Fund, 2021). The global recession due to the pandemic has put an unprecedented strain on government budgets creating risks of diminishing government health expenditure and reducing households’ capacity to cover out-of-pocket healthcare expenses (Kurowski *et al.*, 2021). The latter is of particular concern for lower income countries, where healthcare is financed to a much larger extent through out-of-pocket expenditure. A slower pace of recovery in lower income countries means that those risks will persist there longer and that the disparity between richer and poorer countries in financial barriers to accessing care will likely increase. Evidence from the global financial crisis of 2007–09 confirms this risk. In the USA and in Europe, the prevalence of foregone care increased during the recession and remained high during the immediate recovery period (Elstad, 2016; Towne *et al.*, 2017).

It is likely that a substantial proportion of households in LMICs were foregoing healthcare due to financial and other reasons before the pandemic. However, it is difficult to compare our estimates with pre-pandemic figures. Many of the face-to-face surveys from which the phone surveys samples we made use of were drawn did not collect information on foregone care. Those that did collect information on foregone care used different methodological approaches (e.g. collected data in person from all household members, compared to the phone surveys collecting information about the households from a single respondent) which prevents a direct comparison. The difference in the methodology means observed differences in foregone care prevalence may be attributable to both the broader impact of the COVID-19 crisis and the particulars of the measurement approach, and disentangling the contribution of these is not possible.

In general, in contrast to robust estimates of catastrophic and impoverishing health expenditure (WHO, 2020c), the literature on foregone care in LMICs is remarkably limited. Although many studies have examined foregone care in high-income countries, there are no published analyses of pooled cross-regional and cross-country data from LMICs. Murphy and colleagues provided estimates of foregoing medications for non-communicable diseases due to financial reasons in 18 high-, middle- and low-income countries (Murphy *et al.*, 2020). Although these estimates are not directly comparable with our study, they showed a similar pattern: the proportion of households reporting foregoing medication for financial reasons was higher in low-income countries and gradually declined in the higher income groups. A study synthesizing data from 20 African countries collected in 2008 and 2009 showed that between 35% (in Ghana) and 80% (in Zimbabwe) household went at least once in the 12 months preceding the data collection without either health services or medicines they needed (Abiola *et al.*, 2011). These estimates are substantially higher than the ones reported here, but they use a different recall period (12 months) and combine health services and medicine purchases. Three other published single-country studies from Africa (Ethiopia, Kenya and Liberia) estimated the prevalence of foregone care using different methodological approaches (Mebratie *et al.*, 2014; Bonfrer and Gustafsson-Wright 2017; Gabani and Guinness 2019). A study from 27 countries in the Americas using data spanning 2000–18 estimated the average prevalence of foregone care to be 29.3% for any health services and 16.7% for care for child illnesses (Báscolo *et al.*, 2020). However, foregone care was defined as not seeking care or seeking care that was not deemed to be appropriate. A study using International Social Survey Program data collected in 2011–13 in 23 countries included estimates of foregone care in two upper-middle-income countries: South Africa and Turkey (13.5% and 28.0%, respectively) (Kim *et al.*, 2017). Finally, two published studies have estimated foregone care in China—one among older adults with chronic diseases and another among cancer survivors (Li *et al.*, 2018; Su *et al.*, 2020).

This paper focuses on country-level analysis, comparing differences in foregone care across different country income groups and regions of the world to emphasize the heterogeneity in the reasons behind foregone care and to call attention to the need of potentially different policy responses during the recovery in poorer and richer countries. Future analyses will explore within country differences and the extent to which different household-level characteristics (e.g. household income) affect the likelihood of households’ foregoing care they need.

One potential limitation of this study is that it was based on phone survey data which excluded respondents who did not have access to a phone. This method of data collection was necessary to collect information quickly during the COVID-19 pandemic, while respecting local movement restrictions and minimizing the risk of COVID transmission. Given that having phone may be non-random, this might have created a risk that the results are representative only of the population with access to phones. However, in the countries in this sample, access to mobile phones was generally high—the population-weighted average mobile cellular subscriptions were 99.8 per 100 people in 2019 (World Bank, 2021). Furthermore, sampling weights were used in the analysis to correct for the biases resulting from non-random access to phones. A recent analysis using the same phone survey samples we make use of from four African countries demonstrated that the weighting procedures successfully minimized the selection bias in the phone surveys for a wide range of indicators (Ambel *et al.*, 2021).

Another potential limitation is that the estimates are based on self-reports. This approach is widely used in foregone care studies but poses a risk of recall, response and other types of bias for which alternatives have been proposed (Moreno-Serra *et al.*, 2011; Mebratie *et al.*, 2014; Gabani and Guinness 2019). Mebratie and colleagues used clinical vignettes to assess whether households in Ethiopia used appropriate health services; Gabani and Guiness measured foregone care by estimating the proportion of household who faced a health shock but reported only very small expenditure that was deemed insufficient to obtain the necessary care (Mebratie *et al.*, 2014; Gabani and Guinness 2019). However, collecting data using such methods is more challenging and requires judgement calls from researchers, and, to date, there has been no empirical analysis of the performance of such measures vis-a-vis self-reports.

Finally, the data were collected from one respondent per household reporting for the entire household, rather than from each household member individually. It is possible that the respondent (usually the household head) may not have accurately recalled whether a household member needed care, whether they were not able to access it and why. It is unclear how this could affect the reported reasons for foregoing care. It seems more likely that this source of bias would result in the underestimation of the prevalence of foregone care if the respondent did not recall or was not aware of household members needing and/or not being able to access care (e.g., a male head of household might not have been aware of female household members foregoing reproductive health services). Therefore, the estimates presented here could be considered a likely lower-bound of foregone care.

In sum, the key implication of our paper is that the COVID-19 pandemic has exacerbated old, well-entrenched risks for foregoing healthcare in LMICs and created new ones. With the roll-out of national vaccination programmes, the fear of contracting COVID-19 is expected to diminish, and lockdown measures are already being lifted in countries that have achieved high vaccination coverage. Economic recovery, however, will take longer, and current projections suggest that income inequality between countries will increase in the aftermath of the pandemic. Likewise, our analyses suggest that disparities in access to healthcare may also increase. Therefore, to protect progress towards universal health coverage achieved to date in LMICs, in addition to COVID-19 vaccination, policy actions at the national and global level that ensure financial access to essential health services need to be a critical part of the recovery.

## Supplementary Material

czac024_Supp

## Data Availability

The country-level data underlying this article are available in the COVID-19 Household Monitoring Dashboard at https://www.worldbank.org/en/data/interactive/2020/11/11/covid-19-high-frequency-monitoring-dashboard.
