# Supplementary material for: Foregone healthcare during the COVID-19 pandemic: early survey estimates from 39 low- and middle-income countries
Source: Health Policy Plan. 2022 Mar 11;37(6):771–8. doi: 10.1093/heapol/czac024 (PMC8992243; doi:10.1093/heapol/czac024)
Supplement: czac024_Supp [file czac024_supp.zip › supplementary.docx]

**Annex Table 1: Surveys included in sample**

| Country | Month | Income group | Region |
| --- | --- | --- | --- |
| Argentina | June | Upper middle income | Latin America & Caribbean |
| Burkina Faso | June | Low income | Sub-Saharan Africa |
| Bolivia | June | Lower middle income | Latin America & Caribbean |
| Congo, Dem. Rep. | June | Low income | Sub-Saharan Africa |
| Colombia | June | Upper middle income | Latin America & Caribbean |
| Costa Rica | May | Upper middle income | Latin America & Caribbean |
| Djibouti | July | Lower middle income | Middle East & North Africa |
| Dominican Republic | May | Upper middle income | Latin America & Caribbean |
| Ecuador | June | Upper middle income | Latin America & Caribbean |
| Ethiopia | June | Low income | Sub-Saharan Africa |
| Ghana | June | Lower middle income | Sub-Saharan Africa |
| Guatemala | May | Upper middle income | Latin America & Caribbean |
| Honduras | June | Lower middle income | Latin America & Caribbean |
| Indonesia | May | Upper middle income | East Asia & Pacific |
| Iraq | August | Upper middle income | Middle East & North Africa |
| Cambodia | May | Lower middle income | East Asia & Pacific |
| Lao PDR | July | Lower middle income | East Asia & Pacific |
| St. Lucia | May | Upper middle income | Latin America & Caribbean |
| Madagascar | June | Low income | Sub-Saharan Africa |
| Mexico | June | Upper middle income | Latin America & Caribbean |
| Mali | June | Low income | Sub-Saharan Africa |
| Mongolia | May | Lower middle income | East Asia & Pacific |
| Mozambique | June | Low income | Sub-Saharan Africa |
| Malawi | June | Low income | Sub-Saharan Africa |
| Nigeria | June | Lower middle income | Sub-Saharan Africa |
| Peru | June | Upper middle income | Latin America & Caribbean |
| Philippines | August | Lower middle income | East Asia & Pacific |
| Papua New Guinea | June | Lower middle income | East Asia & Pacific |
| Paraguay | June | Upper middle income | Latin America & Caribbean |
| Senegal | May | Lower middle income | Sub-Saharan Africa |
| El Salvador | June | Lower middle income | Latin America & Caribbean |
| Somalia | July | Low income | Sub-Saharan Africa |
| South Sudan | June | Low income | Sub-Saharan Africa |
| Chad | June | Low income | Sub-Saharan Africa |
| Tunisia | May | Lower middle income | Middle East & North Africa |
| Uganda | June | Low income | Sub-Saharan Africa |
| Vietnam | June | Lower middle income | East Asia & Pacific |
| Zambia | June | Lower middle income | Sub-Saharan Africa |
| Zimbabwe | June | Lower middle income | Sub-Saharan Africa |

**Annex Table 2: Prevalence of specific reasons for foregoing care**

|  | **Lack of money** | **Lack of transportation** | **Afraid/ concerned about catching COVID** | **Movement restrictions (stay–at–home orders)** | **No medical personnel available** | **Facility was full** | **Facility was closed** | **Hospital/clinic did not have enough supplies or tests** | **Other** |
| --- | --- | --- | --- | --- | --- | --- | --- | --- | --- |
|  | **Percentage of households not accessing care** | **Percentage of households not accessing care** | **Percentage of households not accessing care** | **Percentage of households not accessing care** | **Percentage of households not accessing care** | **Percentage of households not accessing care** | **Percentage of households not accessing care** | **Percentage of households not accessing care** | **Percentage of households not accessing care** |
| **All countries** |  |  |  |  |  |  |  |  |  |
| All Countries | 25·6(23·1–28·2) | 6·1(4·5–7·8) | 15·4(13·2–17·6) | 10·0(8·0–12·1) | 18·7(16·2–21·3) | 1·7(1·0–2·4) | 0·3(0·1–0·4) | 0·4(0·1–0·7) | 22·3(19·6–25·0) |
| **Country income group** |  |  |  |  |  |  |  |  |  |
| Low Income | 56·9(51·2–62·7) | 9·5(6·4–12·7) | 5·1(2·6–7·6) | 2·9(0·6–5·2) | 13·6(9·3–17·9) | 4·1(1·5–6·7) | 0·4(–0·1–0·9) | 1·6(0·1–3·1) | 8·1(5·1–11·0) |
| Lower middle– income | 24·5***(20·6–28·3) | 6·6(3·6– 9·6) | 18·2***(14·5–21·8) | 12·2***(8·9–15·5) | 19·2*(15·1–23·3) | 1·8(0·8–2·9) | 0·0(..) | 0·0(..) | 18·3***(14·3–22·3) |
| Upper middle– income | 13·2***(9·3–17·2) | 4·1***(2·1–6·2) | 16·6***(12·8–20·3) | 10·6***(7·1–14·1) | 20·5**(16·4–24·6) | 0·6*(0·0–1·1) | 0·5(0·1–1·0) | 0·3(–0·0–0·6) | 33·3***(28·4–38·2) |
| **Region** |  |  |  |  |  |  |  |  |  |
| East Asia & Pacific | 41·0(33·5–48·6) | 2·5***(0·4–4·7) | 29·4(21·9–36·9) | 3·8(1·4–6·1) | 3·8***(1·2–6·4) | 4·0(1·6–6·4) | 0·0(..) | 1·7(–0·2–3·6) | 13·7(8·3–19·0) |
| Latin America & Caribbean | 6·4***(3·1–9·7) | 6·0(3·8–8·2) | 19·8(15·9–23·7) | 12·1**(8·8–15·5) | 24·6***(20·5–28·7) | 0·0***(0·0– 0·0) | 0·0(..) | 0·0(..) | 31·0***(26·4–35·7) |
| Middle East & North Africa | 27·8***(21·7–34·0) | 0·0(..) | 5·9**(3·0–8·9) | 19·8***(13·3–26·3) | 24·6***(17·9–31·4) | 2·0(–0·1–4·1) | 2·1(0·5–3·7) | 0·0(..) | 17·3(12·4–22·2) |
| Sub–Saharan Africa | 44·3(39·3–49·3) | 8·4(5·0–11·7) | 10·4(7·2–13·6) | 6·3(3·2–9·4) | 13·1(9·1–17·2) | 3·2(1·6–4·9) | 0·2(–0·1–0·4) | 0·7(0·0–1·3) | 15·0(11·0–18·9) |

**Notes:** Data are from World Bank’s High Frequency Phone Surveys fielded between May and August of 2020. Sample is restricted to households reporting indicating some healthcare need during survey’s recall period. Prevalence of foregone care is the proportion of households who report needing care but not accessing needed care. Financial reasons are: lack of money and lack of transportation. COVID reasons are: fear of COVID, and movement restrictions. Supply reasons are: lack of medical personnel, lack of supplies/medication or facility closed/full. Totals of columns (1) – (9) may not add up to 100% exactly due to rounding and as 3 surveys allowed respondents to indicate more than one reason for not accessing care. Large sample z-tests of proportion equality between (i) low income country households and others, and (ii) Sub-Saharan African country households and others respectively, are indicated by stars, with p <0.01 ***, p<0.05 **, and p <0.10

**Weighting-adjustment for pooled**
